# Initial supplementary oxygen concentration for moderate-late preterm infants receiving respiratory support in the delivery room: statistical analysis plan for the multicenter, cluster-randomized crossover AIROPLANE Trial

**DOI:** 10.1186/s13063-026-09437-5

**Published:** 2026-01-14

**Authors:** Stacey Peart, Brett J. Manley, Cecilia Moore, Jeanie L. Y. Cheong, Ju Lee Oei, Li Huang, Peter G. Davis, Louise S. Owen, Anneke Grobler

**Affiliations:** 1 Newborn Services and Newborn Research, Royal Women’s Hospital, Melbourne, Australia; 2 Department of Obstetrics, Gynaecology and Newborn Health, The University of Melbourne, Melbourne, Australia; 3 Clinical Sciences, Murdoch Children’s Research Institute, Melbourne, Australia; 4 Mercy Perinatal, Mercy Hospital for Women, Melbourne, Australia; 5 Clinical Epidemiology and Biostatistics Unit, Murdoch Children’s Research Institute, Melbourne, Australia; 6 Department of Paediatrics, The University of Melbourne, Melbourne, Australia; 7 National Health and Research Medical Council Clinical Trials Centre, The University of Sydney, New South Wales, Australia; 8 Department of Newborn Care, The Royal Hospital for Women, Randwick, NSW Australia; 9 Discipline of Paediatrics and Child Health, School of Clinical Medicine, University of New South Wales, Kensington, New South Wales, Australia; 10 Melbourne School of Population and Global Health, The University of Melbourne, Melbourne, Australia

## Abstract

**Background:**

Moderate-late preterm infants born at 32–35 completed weeks’ gestation constitute a large proportion of all preterm births (< 37 weeks’ gestation), yet they are not well represented in the newborn resuscitation literature. Preterm infants often receive respiratory support in the delivery room, and recommendations exist to guide the use of supplemental oxygen when providing this support. However, there are minimal data regarding the best initial supplementary oxygen concentration for moderate-late preterm infants requiring respiratory support at birth, resulting in practice variation. The aim of the AIROPLANE trial is to compare initial supplementary oxygen concentrations of 30% and 21% (air) in preterm infants of 32–35 weeks’ gestation who require respiratory support in the delivery room, with a primary outcome of the need for ongoing respiratory support upon leaving the delivery room.

**Methods:**

A prospective, unblinded, multicenter, cluster-randomized, crossover trial in Australian maternity hospitals comparing initial supplementary oxygen concentrations of 30% and 21% (air) in moderate-late preterm infants who require respiratory support at birth. Eligible infants are those born from 32 + 0 to 35 + 6 weeks’ gestation without major cardiorespiratory or craniofacial anomalies, who are receiving active care, and who receive respiratory support in the delivery room commencing within the first three minutes after birth. The primary outcome is the need for ongoing respiratory support upon leaving the delivery room. The trial will recruit a minimum of 1,200 infants from at least 20 study sites in Australia using a waiver of consent process.

**Statistical analysis plan:**

To be able to demonstrate an absolute reduction in the primary outcome of 8% (relative reduction 16%), from 51 to 43%, with 80% power and 5% alpha level, a minimum sample of 1200 infants from at least 20 participating sites (20 clusters) with one crossover halfway through each site’s total enrolment period is required. The primary outcome, whether the infant is receiving respiratory support when leaving the delivery room, will be analyzed as a binary outcome. The incidence of this outcome will be summarized as the number and percentage in each treatment arm. The treatments will be compared using a risk difference and 95% confidence interval (CI) using individual participant level data. A cross-sectional sample in each treatment period will be modelled with a mixed effects generalized linear model (GLM) with an identity link function and a binomial distribution using an exchangeable correlation structure to model the correlation within each cluster, adjusted for treatment period due to the cross-over nature. The primary analysis will be by intention-to-treat.

**Trial registration:**

ACTRN12621001267842. Registered on 20th September 2021. Australian New Zealand Clinical Trials Registry (https://www.anzctr.org.au).

## Introduction

With rates of preterm birth (< 37 weeks’ gestation) increasing [1], newborn resuscitation remains a critical area of neonatal research. International guidelines have continued to evolve over the last two decades, reflecting the evidence most widely noted in term infants and in extremely preterm infants (< 28 weeks’ gestation). However, moderate-late preterm infants, born from 32 weeks to 36 completed weeks’ gestation, are not well represented in newborn resuscitation literature despite this cohort constituting the majority of preterm births [2] (https://www.aihw.gov.au/reports/mothers-babies/australias-mothers-and-babies-2018-in-brief/data). This has resulted in a lack of evidence-based resuscitation guidelines for this group.

The International Liaison Committee on Resuscitation (ILCOR) currently recommends commencing respiratory support at birth with 21% oxygen (air) for infants ≥ 35 weeks’ gestation [3]. However, the concentration of supplemental oxygen for commencing respiratory support at birth in preterm infants remains unclear, with contrasting international guidelines for infants born 32–35 weeks’ gestation [4] (https://www.aihw.gov.au/reports/mothers-babies/australias-mothers-babies/contents/baby-outcomes/gestational-age). To date, no study has specifically addressed the question of optimal initial oxygen concentration when commencing respiratory support at birth in infants born 32–35 weeks’ gestation.

## Study synopsis

The AIROPLANE Trial is a prospective, unblinded, multicenter, superiority, cluster-randomized crossover trial comparing initial oxygen concentrations of 30% and 21% (air) in infants born between 32 + 0 and 35 + 6 weeks’ gestation, who receive respiratory support at birth. The primary outcome is the need for ongoing respiratory support upon leaving the delivery room. The trial will recruit for 32 months, in a minimum of 20 Australian maternity hospitals, to enroll a minimum of 1,200 infants in total. Full details of the trial are available in the published protocol [5].

### Primary objective

To determine, in infants born between 32 + 0 and 35 + 6 weeks’ gestation who receive respiratory support at birth, whether commencing respiratory support with 30% supplemental oxygen, or 21% oxygen (air), reduces the need for ongoing respiratory support upon leaving the delivery room.

### Secondary objectives

Secondary outcomes collected at the time of leaving the delivery room and/or at the time of hospital discharge include:

- Apgar scores at 1 and 5 min of age
- Delivery room resuscitation interventions; these include use of the following: (1) non-invasive respiratory support (CPAP or nasal high-flow), (2) IPPV via a non-invasive interface, (3) endotracheal intubation or supraglottic airway insertion, (4) cardiac compressions and/or epinephrine administration, and (5) maximum fraction of inspired oxygen (FiO_2_).
- Age (in days) at final day of ongoing respiratory supports beyond the delivery room (including final day of mechanical ventilation, of non-invasive respiratory support, of supplemental oxygen, and of any respiratory support)
- Treatment with exogenous surfactant
- Duration of hospitalization (in days)
- Inter-hospital transfers due to escalating care requirements
- Death before hospital discharge.

### Study population

Infants born between 32 + 0 and 35 + 6 weeks’ gestation at a participating maternity hospital who meet the following criteria:

## Inclusion criteria (all must apply)

- a. Receiving active care
- b. Receive respiratory support in the first 3 min after birth

## Exclusion criteria

- a. An antenatal diagnosis of a major cardiorespiratory or craniofacial anomaly

### Intervention

The use of 30% oxygen or 21% oxygen (air) when providing respiratory support at birth to infants born between 32 + 0 and 35 + 6 weeks’ gestation. A detailed explanation of the trial intervention is presented in the published protocol [5].

### Randomization and blinding

#### Randomization

The AIROPLANE Trial is a cluster-randomized, crossover trial in which hospitals are randomized (rather than individual infants). Once a hospital’s eligibility and participation has been confirmed, the hospital is randomized using a web-based application. The randomization schedule is prepared by an independent statistician not directly involved in the study. Hospitals are cluster randomized in a 1:1 ratio using simple block randomization. There will be random allocation to the order in which the two treatments of the trial will be assigned at each site. For half of the study recruitment period at each hospital, that hospital will be assigned to use either 30% oxygen or 21% oxygen (air) for all stabilizations in the delivery room for all eligible infants. The cluster crossover design necessitates that each site provides each study treatment for the same duration of time (e.g., 6 months using 21% oxygen, then 6 months using 30% oxygen). The total study duration will differ between sites according to when each site commenced recruitment. Total study duration at each site will be calculated from the date of activation of that site, to the overall end date of the trial, with the total duration split into two equal durations for each study treatment.

Once deemed eligible by meeting all inclusion criteria and none of the exclusion criteria, participants will receive the allocated treatment that the site has been randomized to during that period (30% oxygen or 21% oxygen).

### Blinding

This is an unblinded study. Clinicians will be aware of the allocated treatment arm, and they will set and adjust the allocated FiO_2_. The central study team, Trial Steering Committee (TSC) and Data and Safety Monitoring Committee (DSMC), will not be blinded to site randomization.

### Sample size

Based on pre-trial data from the lead center (The Royal Women’s Hospital, Melbourne, Australia), approximately 51% of infants born between 32 + 0 and 35 + 6 weeks’ gestation who receive respiratory support at birth continue to receive respiratory support after leaving the delivery room.

A minimum sample size of 1200 infants, from at least 20 sites (clusters), is required to demonstrate an absolute reduction in the primary outcome (need for respiratory support upon leaving the delivery room) of 8% (relative reduction 16%) from 51% to 43%, with 80% power and a 5% alpha level, two-sided. We assumed an average cluster size of 30 per period, a coefficient of variation of 4, an intracluster correlation coefficient (ICC) of 0.02, and an exchangeable correlation structure. The allocation ratio was 1:1, with two periods. The sample size was calculated using https://clusterrcts.shinyapps.io/rshinyapp/. No adjustment for loss to follow-up was made. If a larger number of clusters and/or a greater sample size are obtained, the power to demonstrate this difference will be increased.

Due to the cluster-randomized, crossover design of the trial, the study duration is time-based rather than based on the sample size obtained; this will ensure each site is allocated to both treatment arms for an equal duration.

### Study procedures

Full details of the study procedure are presented in the published trial protocol [5].

The trial will be conducted in a minimum of 20 maternity hospitals in Victoria and New South Wales, Australia, comprising a mixture of tertiary metropolitan, non-tertiary metropolitan, and regional centers, including both public and private facilities. Hospital sites, rather than individual participants, will be randomized to one of the two treatments (30% supplemental oxygen or 21% oxygen) for the first half of the study (Period 1), before crossing over to the alternative treatment for the second half of the study (Period 2), lasting the same amount of time. A diagram of the realised design will be provided.

Clinicians determine the need to provide respiratory support in the delivery room. Infants who receive any respiratory support (i.e., CPAP or IPPV, via any interface) commencing within the first three minutes after birth will commence this support using the allocated oxygen concentration.

“Timepoint 1” data collection will occur upon leaving the delivery room. These data are entered by clinicians via a scanned quick response (QR) code linked to a survey and database (™, Vanderbilt University) [6] hosted by the trial Sponsor. Alternatively, data may also be entered directly into REDCap by research staff at participating sites. “Timepoint 2” data collection occurs in the same manner at the time of transfer to another hospital and/or discharge home from hospital.

### Departures from approved protocol

In the protocol, birth weight is listed as a secondary objective. However, birth weight is determined prior to study intervention and is more accurately classified as a participant characteristic; it will therefore be presented as such (Table 2).

Protocol-specified secondary objectives include the details of resuscitation received in the delivery room. Individual components of this outcome are not specified in the protocol, but are described in this SAP and include the use of the following: (1) non-invasive respiratory support (CPAP or nasal high-flow), (2) IPPV via a non-invasive interface, (3) endotracheal intubation or supraglottic airway insertion, (4) cardiac compressions and/or epinephrine administration, and (5) maximum fraction of inspired oxygen (FiO_2_).

### Participant timeline

**Fig. 1:**
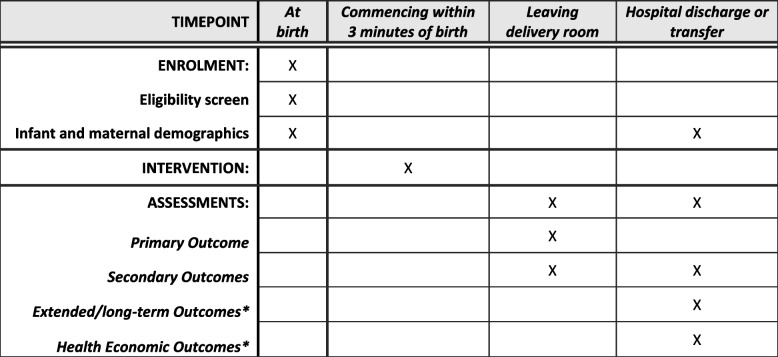
Participant timeline

The participant timeline is shown in Fig. 1.

## General statistical methodology

All analyses will be done at the participant level, adjusted for clustering.

### Objectives of analysis plan

This statistical analysis plan (SAP) covers the analysis of the primary outcome and in-hospital secondary outcomes.

This SAP does not cover planned analyses of:

- Extended secondary outcomes available via data linkage for the subset of infants born in Victoria, Australia, who are consented and co-enrolled into the Generation V (GenV) study.
- Health economic outcomes

These outcomes will be analyzed and reported separately.

### Analysis software

Data will be exported from the study database to Stata (StataCorp. 2019. *Stata Statistical Software:* College Station, TX, USA: StataCorp LLC) for analysis, version 18 or later.

### Data verification

All data will be checked and cleaned prior to analysis. This will include ensuring data validity and completeness. Data quality checks will be run within the REDCap database. Data analysis will commence only after the database is locked and the SAP has been made publicly available.

### Definition of analysis populations

The analysis population will comprise all eligible infants from all randomized sites. The primary analysis will be by intention-to-treat, including all enrolled infants regardless of adherence to the trial protocol. Participants whose parents have opted to have their child’s data removed will be excluded from the analysis.

Data collected during the “washout period,” which is defined as the first week after treatment crossover at each site (at commencement of the second study period and alternative treatment arm), will not be included in the analysis. This pre-planned washout period will minimize cluster-level carryover risk allowing for changeover of all study signage and staff re-education. Eligible infants born in the washout period will receive study treatment and have data collected but not included. Any deaths of infants during this period will be reported separately.

### Adjustment for multiplicity

There will be no adjustment for multiplicity.

### Interim analyses

There will be no interim analysis.

### Handling of missing data

Given the short in-hospital follow-up period, with all primary and secondary data collection aimed to be complete prior to hospital discharge, it is anticipated that there will be 100% ascertainment of the primary outcome, and close to 100% follow-up for secondary outcomes. Every effort will be made to obtain missing data. If there is more than 10% missingness in any outcome, multiple imputation will be used to perform a valid analysis under the “missing at random” assumption.

### Timing of final analysis

Data analysis will commence only after data entry is complete and the database is cleaned and locked, and the SAP has been made publicly available.

### Descriptive statistics

#### Recruitment and follow-up

All infants born 32–35 + 6 weeks’ gestation will be assessed for eligibility for inclusion in the trial.

**Fig. 2:**
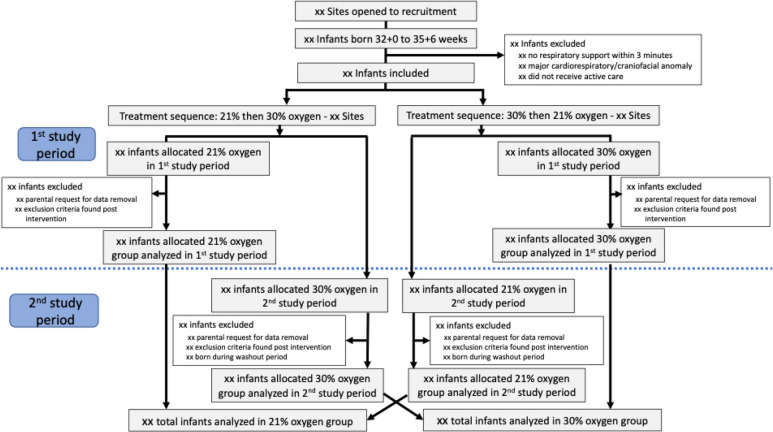
Simplified Consolidated Standards of Reporting Trials (CONSORT) 2010 flow diagram

Figure 2 shows participant flow according to CONSORT reporting for cluster randomized, crossover trials [7].

#### Baseline characteristics

Tables showing baseline, cluster-level characteristics by sequence (Table 1), individual-participant characteristics by sequence period and treatment condition (Table 2), and individual-participant characteristics by treatment condition (Table 3) will be provided. The average cluster size and its variance will be reported [8]. The number of births, number of included infants per period, and sequence by site (cluster) will be provided (Table 4). The number of infants, and any deaths during the washout period will be reported.

**Table 1:** Cluster (site) characteristics

Cluster characteristic	Sequence	
	**Period 1: 21% oxygen** **Period 2: 30% oxygen**	**Period 1: 30% oxygen** **Period 2: 21% oxygen**
**State**		
Victoria New South Wales	*n*(%) *n*(%)	*n*(%) *n*(%)
**Location**		
Metropolitan^ Regional	*n*(%) *n*(%)	*n*(%) *n*(%)
**Funding designation**		
Public hospital Private hospital	*n*(%) *n*(%)	*n*(%) *n*(%)
Level of care capability*		
Level 3 Level 4 Level 5 Level 6	*n*(%) *n*(%) *n*(%) *n*(%)	*n*(%) *n*(%) *n*(%) *n*(%)
Annual number of births in the 32 + 0 to 35 + 6 week’ gestational range	XXX	XXX
Total number of births during the study in the 32 + 0 to 35 + 6 week’ gestational range		

^As defined by Department of Health, Victoria [9] and New South Wales Health [10]

*Level 3 capability describes hospitals that care for infants ≥ 34 weeks’ gestation or birthweight ≥ 2000 g and provide non-invasive respiratory support for up to 72 h; level 4 capability describes hospitals that care for infants ≥ 32 weeks’ gestation or birthweight ≥ 1500 g and provide non-invasive respiratory support for up to 96 h; level 5 capability describes hospitals that care for infants ≥ 31 weeks’ gestation or ≥ 1250 g and provide ongoing non-invasive respiratory support including administration of minimally invasive surfactant therapy; level 6 capability describes specialist care for critically unwell newborns of any gestation or weight [11]. Level of capability for sites in New South Wales has been extrapolated according to Victorian guidelines to ensure consistency in reporting

**Table 2:** Individual participant characteristics per sequence period

Characteristic	Period 1, treatment condition 21% oxygen (*n* = xx)	Period 2, treatment condition 21% oxygen (*n* = xx)	Period 1, treatment condition 30% oxygen (*n* = xx)	Period 2, treatment condition 30% oxygen (*n* = xx)
**Mothers**				
**Mode of delivery**				
Vaginal birth Assisted vaginal delivery Cesarean section	*n*(%) *n*(%) *n*(%)	*n*(%) *n*(%) *n*(%)	*n*(%) *n*(%) *n*(%)	*n*(%) *n*(%) *n*(%)
**Exposure to antenatal corticosteroids**				
None One dose Two or more doses Unknown	*n*(%) *n*(%) *n*(%) *n*(%)	*n*(%) *n*(%) *n*(%) *n*(%)	*n*(%) *n*(%) *n*(%) *n*(%)	*n*(%) *n*(%) *n*(%) *n*(%)
Grouped as:				
Complete antenatal steroids*None/incomplete antenatal steroids	*n*/*N* (%)*n*/*N* (%)	*n*/*N* (%)*n*/*N* (%)	*n*/*N* (%)*n*/*N* (%)	*n*/*N* (%)*n*/*N* (%)
**Infants**				
** Gestational age (weeks)**	Mean (SD)	Mean (SD)	Mean (SD)	Mean (SD)
** Birth weight (grams)**	Mean (SD)	Mean (SD)	Mean (SD)	Mean (SD)
** Male sex**	*n*(%)	*n*(%)	*n*(%)	*n*(%)
** Multiple birth**	*n*(%)	*n*(%)	*n*(%)	*n*(%)
** Delayed cord clamping for at least 60 s**	*n*/*N*(%)	*n*/*N*(%)	*n*/*N*(%)	*n*/*N*(%)

*Two or more doses

**Table 3:** Baseline characteristics of individual participants

Characteristic	Treatment condition	
	**30% oxygen (*n* = XXX)**	**21% oxygen (*n* = XXX)**
**Mothers**		
** Mode of delivery**		
Vaginal birth Assisted vaginal delivery	*n*(%) *n*(%)	*n*(%) *n*(%)
Cesarean section	*n*(%)	*n*(%)
**Exposure to antenatal corticosteroids** None		
One dose	*n*(%)	*n*(%)
Two doses	*n*(%)	*n*(%)
Unknown	*n*(%)	*n*(%)
Grouped as:		
Complete antenatal steroids*None/incomplete antenatal steroids	*n*/*N* (%)*n*/*N* (%)	*n*/*N* (%)*n*/*N* (%)
**Infants**		
** Gestation age (weeks)**	Mean (SD)	Mean (SD)
** Birth weight (grams)**	Mean (SD)	Mean (SD)
** Male sex**	*n*(%)	*n*(%)
** Multiple birth**	*n*(%)	*n*(%)
** Delayed cord clamping for at least 60 s**	*n*/*N*(%)	*n*/*N* (%)

*Two or more doses

**Table 4:** Number of infants included per cluster

Cluster	Total births 32 + 0 to 35 + 6 weeks’ GA during 30% oxygen period	Included infants during 30% oxygen period	Total births 32 + 0 to 35 + 6 weeks’ GA during 21% oxygen period	Included infants during 21% oxygen period	Sequence
Site 1	*n*	*n*(%)	*n*	*n*(%)	
Site 2	*n*	*n*(%)	*n*	*n*(%)	
Site 3	*n*	*n*(%)	*n*	*n*(%)	
Site 4	*n*	*n*(%)	*n*	*n*(%)	
Site 5	*n*	*n*(%)	*n*	*n*(%)	
Site 6	*n*	*n*(%)	*n*	*n*(%)	
Site 7	*n*	*n*(%)	*n*	*n*(%)	
Site 8	*n*	*n*(%)	*n*	*n*(%)	
Site 9	*n*	*n*(%)	*n*	*n*(%)	
Site 10	*n*	*n*(%)	*n*	*n*(%)	
Site 11	*n*	*n*(%)	*n*	*n*(%)	
Site 12	*n*	*n*(%)	*n*	*n*(%)	
Site 13	*n*	*n*(%)	*n*	*n*(%)	
Site 14	*n*	*n*(%)	*n*	*n*(%)	
Site 15	*n*	*n*(%)	*n*	*n*(%)	
Site 16	*n*	*n*(%)	*n*	*n*(%)	
Site 17	*n*	*n*(%)	*n*	*n*(%)	
Site 18	*n*	*n*(%)	*n*	*n*(%)	
Site 19	*n*	*n*(%)	*n*	*n*(%)	
Site 20	*n*	*n*(%)	*n*	*n*(%)	
Site 21	*n*	*n*(%)	*n*	*n*(%)	
Site 22	*n*	*n*(%)	*n*	*n*(%)	
Site 23	*n*	*n*(%)	*n*	*n*(%)	
Site 24	*n*	*n*(%)	*n*	*n*(%)	
Site 25	*n*	*n*(%)	*n*	*n*(%)	
Site 26	*n*	*n*(%)	*n*	*n*(%)	
**Average**	***n***	***n*(%)**	***n***	***n*(%)**	
**Standard deviation**	**-**	**-(-)**	**-**	**-(-)**	

#### Protocol deviations

A major protocol deviation is defined as:

- Commencing respiratory support with any FiO_2_ other than that allocated.

A minor protocol deviation is defined as:

- Adjusting the allocated FiO_2_ prior to three minutes after birth, or prior to provision of one minute of respiratory support (whichever timepoint is later), unless also providing cardiac compressions or endotracheal intubation.

Protocol deviations will not result in the exclusion of participants from the analysis. An intention-to-treat analysis will be undertaken with no plan for a per-protocol analysis.

#### Compliance

Data regarding study compliance will be collected on the initial Case Report Form, completed for each infant immediately following administration of the study treatment. These data include the initial FiO_2_ used when providing respiratory support, and any changes made to the FiO_2_ within three minutes of birth, or prior to provision of one minute of respiratory support, if later.

Compliance will be summarized as the number and proportion of infants in each treatment condition who received the intervention as randomized. In those where changes were made to randomly allocated FiO_2_ within three minutes of birth, or prior to the provision of one minute of respiratory support, the number and proportion where this was the result of a protocol deviation (as opposed to a clinical indication) will be reported.

## Analysis of the primary outcome

### Main analysis

The primary outcome, whether the infant was receiving respiratory support when leaving the delivery room, will be analyzed as a binary outcome at the participant level. This cluster-crossover trial collected cross-sectional data, with no repeated measures on the same participant, either in the same period or in the crossover period. The incidence of this outcome will be summarized as the number and percentage in each treatment condition. Carryover effects of the crossover design will not be assessed.

#### Estimand for the primary outcome

##### Population

Infants born in a participating hospital between 32 + 0 and 35 + 6 weeks’ gestation, who receive respiratory support in the delivery room.

##### Treatment

Initial use of 30% supplemental oxygen or 21% oxygen (air) when receiving respiratory support in the delivery room within 3 min of birth.

##### Outcome

Whether the infant is receiving respiratory support when leaving the delivery room (binary yes/no outcome). Respiratory support is defined as any of the following: positive pressure ventilation via endotracheal tube or supraglottic airway, any form of non-invasive respiratory support (such as CPAP using a facemask, nasal mask, bi-nasal prongs, or single nasal prong, or nasal high flow). Provisions of supplemental oxygen only, without positive pressure, are not considered as receiving respiratory support.

##### Summary measure

Risk difference and 95% confidence interval (CI).

##### Potential intercurrent events

- Commencing respiratory support with any FiO_2_ other than that allocated.
- Adjusting the FiO_2_ prior to three minutes after birth, or prior to provision of 1 min of respiratory support (unless providing cardiac compressions or endotracheal intubation).
- Death prior to leaving the delivery room

##### Handling of intercurrent events

The intercurrent events will be handled using the treatment policy strategy, which means that the observed data will be used in the analysis regardless of whether the intercurrent events occurred, except for the intercurrent event of death prior to leaving the delivery room, in which case the composite strategy will be used. This means that infants who died in the delivery room will be regarded as having a negative outcome (i.e. were receiving respiratory support on leaving the delivery room).

The type of respiratory support received when leaving the delivery room will be summarized by number and percentage of infants in each of the categories.

The treatment conditions will be compared using a risk difference, risk ratio, and 95% CI using individual participant level data. A cross-sectional sample in each sequence period will be modelled with a hierarchical generalized linear mixed model (GLMM) with an identity link function and a binomial distribution to fit a binomial model, with ‘receiving respiratory support’ as the outcome, and cluster (site), with infants who are part of the same multiple birth modelled as random effects. Treatment condition and period (before crossover vs. after crossover) will be considered fixed effects. An interaction between period and treatment will be used to evaluate whether differences between periods were affected by treatment; an overall effect of the treatment by period interaction will be presented.

If the model does not converge, the same model will be fitted with a logistic link and the risk difference estimated by calculating the predicted probabilities (risks) for each treatment group using the margins command. The difference between these predicted risks will provide the marginal risk difference, along with CIs and p-values.

In addition to the risk difference, the risk ratio will be calculated comparing the two treatment arms, using the same model as for the risk difference, except by including a log rather than a linear link function.

### Sensitivity analyses

A sensitivity analysis will be performed by fitting the same model, adjusted for study site, antenatal corticosteroid exposure, mode of delivery, gestational age, and birth weight. If collinearity makes it impossible to fit a model with all these covariates, the most correlated covariates will be excluded.

### Subgroup analyses

Subgroup analyses will be performed by gestational age (32 + 0 to 33 + 6 weeks’ vs. 34 + 0 to 35 + 6 weeks’ gestation), and by hospital capability level (levels 3–5 vs level 6).

The primary outcome, detailed components, sensitivity analyses, and sub-group analyses will be provided (Tables 5 and 6).

**Table 5:** Primary outcome by treatment arm

Primary outcome	21% oxygen *N *=	30% oxygen *N* =				
**Respiratory support on leaving the delivery room, *N*(%)**			**Risk difference**	***p*-value** **(95% CI)**	**Risk ratio**	***p*-value** **(95% CI)**
Any respiratory support* or deceased	XXX (XX%)	XXX (XX%)	X.XX	X.X (X, X)	X.XX	X.X (X, X)
Sensitivity analysis**	XXX (XX%)	XXX (XX%)	X.XX	X.X (X, X)	X.XX	X.X (X, X)
**Type of respiratory support on leaving the delivery room**						
No respiratory support, in room air	XXX (XX%)	XXX (XX%)				
No respiratory support, in supplemental oxygen	XXX (XX%)	XXX (XX%)				
Respiratory support with CPAP or High Flow (via face-mask, nasal mask or nasal prong/s)	XXX (XX%)	XXX (XX%)				
Ongoing positive pressure ventilation (via facemask or supraglottic airway)	XXX (XX%)	XXX (XX%)				
Intubated with an endotracheal tube	XXX (XX%)	XXX (XX%)				
Deceased	XXX (XX%)	XXX (XX%)				

*Any respiratory support includes CPAP or high flow, intubation, or ongoing intermittent positive pressure ventilation

**Fully adjusted analysis, including adjustment for study site, antenatal corticosteroid exposure, mode of delivery, gestational age and birth weight

**Table 6:** Subgroup analysis for primary endpoint

Subgroup analyses	21% oxygen *N* (%)	30% oxygen *N*(%)	Risk difference (95% CI)	*p*-value**	Risk ratio (95% CI)	*p*-value**
**Gestational age: 32 + 0 to 33 + 6 weeks**				xx		xx
Any respiratory support* or deceased	XXX (XX%)	XXX (XX%)	X.XX (X, X)		X.XX (X, X)	
**Gestational age: 34 + 0 to 35 + 6 weeks**						
Any respiratory support* or deceased	XXX (XX%)	XXX (XX%)	X.XX (X, X)		X.XX (X, X)	
**Hospital capability level: 3–5**				xx		xx
Any respiratory support* or deceased	XXX (XX%)	XXX (XX%)	X.XX (X, X)		X.XX (X, X)	
**Hospital capability level: 6**						
Any respiratory support* or deceased	XXX (XX%)	XXX (XX%)	X.XX (X, X)		X.XX (X, X)	

*Any respiratory support includes CPAP or high flow, intubation, or ongoing intermittent positive pressure ventilation

**p-value is the interaction of treatment by period

## Secondary outcomes

Secondary outcomes will be analyzed as outlined in the “General statistical methodology” section, as shown in Table 7.

**Table 7:** In-hospital secondary outcomes

Outcome	21% oxygen (*n* =)	30% oxygen (*n* =)	Risk/mean/median difference (95% CI)	*p*-value	Risk/common odds ratio (95% CI)	*p*-value
**Apgar score (median, 95% CI)**						
1 min	X (X, X)	X (X, X)	X (95% CI)	X.XX	X (95%CI)	X.XX
5 min	X (X, X)	X (X, X)	X (95% CI)	X.XX		X.XX
**Highest level of support in the delivery room**					**Odds ratio**	
Non-invasive respiratory support (CPAP or nasal high flow)	*n*(%)	*n*(%)			X (95% CI)	XX
IPPV via a non-invasive interface Endotracheal Intubation or SGA insertion with IPPV Cardiac compressions and/or epinephrine	*n*(%) *n*(%) *n*(%)	*n*(%) *n*(%) *n*(%)				
**Maximum FiO**_**2**_** in the delivery room**	Mean (95% CI)	Mean (95%CI)	X (95% CI)	X.XX		
**Ongoing respiratory support beyond the delivery room**						
Age at final day of intubation and mechanical ventilation (days)	*n*(%)median(95% CI)	n(%)median(95% CI)	X (X-X)	X.XX	X (X-X)	X.XX
Age at final day of non-invasive respiratory support	*n*(%)Median(95% CI)	*n*(%)Median(95% CI)	X (X-X)	X.XX	X (X-X)	X.XX
Age at final day of supplemental oxygen (days)	*n*(%)Median(95% CI)	*n*(%)Median(95% CI)	X (X-X)	X.XX	X (X-X)	X.XX
Age at final day of any respiratory support and/or supplemental oxygen (days)	*n*(%) Median (95% CI)	*n*(%) Median (95% CI)	X (X-X)	X.XX	X (X-X)	X.XX
**Received exogenous surfactant**	*n*(%) (95% CI)	*n*(%) (95%CI)	X (X-X)	X.XX	X (X-X)	X.XX
**Duration of hospital admission (days)**	Mean (95% CI) Median (95% CI)	Mean (95% CI) Median (95% CI)	X (X-X) X (X-X)	X.XX		
**Interhospital transfer due to escalating care requirements**	*n*(%)	*n*(%)	X (X-X)	X.XX	X (X-X)	X.XX
**Death before hospital discharge**	*n*(%)	*n*(%)				
**Death in washout period**	*n*(%)	*n*(%)				

*FiO*_*2*_, fraction of inspired oxygen; *IPPV*, intermittent positive pressure ventilation; *CPAP*, continuous positive airway pressure; *SGA*, supraglottic airway; *ETT*, endotracheal tube; *CI*, confidence interval

### Apgar scores at 1 and 5 min of age

Apgar scores will be analyzed as a continuous variable with the median reported in each treatment condition. The treatment conditions will be compared using the difference in medians (with 95% CI) using individual participant level data. A cross-sectional sample in each sequence period will be modelled with a linear quantile mixed model, with Apgar score as the outcome and cluster (hospital) and multiple birth modelled as random effects. Treatment and period (before crossover vs. after crossover) will be considered fixed effects. An interaction between period and treatment will be used to evaluate whether differences between periods were affected by treatment. (This model cannot be fitted in Stata, and the R lqmm command will be used to fit the model).

### Delivery room resuscitation interventions

The highest level of respiratory support provided in the delivery room will be presented as an ordinal outcome: (1) non-invasive respiratory support (CPAP or nasal high-flow), (2) IPPV via a non-invasive interface, (3) endotracheal intubation or supraglottic airway insertion with IPPV, (4) cardiac compressions and/or epinephrine administration. This will be summarized by giving the number and percentage of infants in each category by treatment arm. A mixed effects proportional odds logistic regression will be used to calculate the overall odds ratio. Cluster (hospital) and infants who are part of the same multiple birth modelled as random effects, and treatment condition and period (before crossover vs. after crossover) will be considered fixed effects. The assumption of proportional odds will be investigated. If it is violated, a multinomial regression will be fitted and an odds ratio calculated for each category.

### Maximum FiO_2_ in the delivery room

The mean maximum FiO_2_ will be reported as a continuous outcome in each treatment condition. The treatment conditions will be compared using a difference in means and 95% CI using individual participant level data. A cross-sectional sample in each sequence period will be modelled with a GLMM with an identity link function and a Gaussian distribution to fit a linear regression model, with maximum FiO_2_ as the outcome and cluster (hospital) and infants who are part of the same multiple birth modelled as random effects. Treatment condition and period (before crossover vs. after crossover) will be considered fixed effects. An interaction between period and treatment will be used to evaluate whether differences between periods were affected by treatment.

### Ongoing respiratory support beyond the delivery room

Age (in days) at final day of (1) endotracheal intubation and ventilation, (2) non-invasive respiratory support, (3) supplemental oxygen, and (4) any respiratory support (endotracheal intubation or non-invasive respiratory support or supplemental oxygen) will be analyzed. Any part of a calendar day will be designated as 1 day. Infants who did not receive ongoing respiratory support will be regarded as having an age of 0 at final day of respiratory support.

It is anticipated that more than 50% of participants will receive zero days of respiratory support. For this reason, the continuous variable will be converted to a binary variable in order to compare the arms. If age at final day of respiratory support is zero, respiratory support will be coded as absent; if age at final day is above zero, it will be coded as present.

Estimand for presence of ongoing respiratory support beyond the delivery room (including mechanical ventilation, non-invasive respiratory support and/or supplemental oxygen and any respiratory support):

- Population: Infants born in a participating hospital between 32 + 0 and 35 + 6 weeks’ gestation, who receive respiratory support at birth.
- Treatment: Initial use of 30% supplemental oxygen or 21% oxygen (air) in the delivery room regardless of changes after 3 min.
- Outcome: Age at final day of each of the four respiratory support outcomes (difference between date of birth and last day of that mode of respiratory support or supplemental oxygen use). If age at final day of respiratory support/supplemental oxygen is zero, respiratory support will be coded as absent; if age at final day is greater than zero, it will be coded as present.
- Summary measure: Risk difference and 95% CI.
- Potential intercurrent events:
  - Commencing respiratory support in any FiO_2_ other than that allocated.
  - Adjusting the FiO_2_ prior to 3 min after birth, or prior to 1 min of respiratory support (unless providing external cardiac compressions or endotracheal intubation).
- Handling of intercurrent events: The intercurrent events will be handled using the treatment policy strategy, which means that the observed data will be used in the analysis regardless of whether the intercurrent events occurred.
- The treatment conditions will be compared using a risk difference, risk ratio, and 95% CI using individual participant level data. A cross-sectional sample in each sequence period will be modelled with a hierarchical generalized linear mixed model (GLMM) with an identity link function and a binomial distribution to fit a binomial model, with “ongoing respiratory support” as the outcome, and cluster (hospital), and infants who are part of the same multiple birth modelled as random effects. Treatment condition and period (before crossover vs. after crossover) will be considered fixed effects. An interaction between period and treatment will be used to evaluate whether differences between periods were affected by treatment; an overall effect of the treatment by period interaction will be presented.
- If the model does not converge, the same model will be fitted with a logistic link and the risk difference estimated by calculating the predicted probabilities (risks) for each treatment group using the margins command. The difference between these predicted risks will provide the marginal risk difference, along with CIs and *p*-values.
- In addition to the risk difference, the risk ratio will be calculated comparing the two treatment arms, using the same model as for the risk difference, except by including a log rather than a linear link function.
- In addition, the age of final support for each of the four respiratory supports/supplemental oxygen outcomes will be summarized as a median in each of the treatment conditions in individuals who required respiratory support. A cross-sectional sample in each sequence period will be modelled with a linear quantile mixed model, with age of final support as the outcome and cluster (hospital) and multiple birth modelled as random effects. Treatment and period (before crossover vs. after crossover) will be considered fixed effects. An interaction between period and treatment will be used to evaluate whether differences between periods were affected by treatment. This will be provided as a summary of the median (with 95% CI) in each of the treatment conditions only.

### Treatment with exogenous surfactant

This will be analyzed as described in the “Main analysis” section for the primary outcome.

### Hospital admission duration (days)

Duration of hospitalization from birth to first discharge will be calculated in days. Any part of a calendar day will be designated as 1 day.

#### Estimand

##### Population

Infants born in a participating hospital between 32 + 0 and 35 + 6 weeks’ gestation, who receive respiratory support at birth.

##### Treatment

Initial use of 30% supplemental oxygen or 21% oxygen (air) in the delivery room regardless of changes after 3 min

##### Outcome

Duration of hospitalization

##### Summary measure

Mean and median difference in duration of hospitalization, with 95% CI and p-value.

##### Potential intercurrent events

- Commencing respiratory support in any FiO_2_ other than that allocated.
- Adjusting the FiO_2_ prior to 3 min after birth, or prior to 1 min of respiratory support (unless providing external cardiac compressions or endotracheal intubation).

##### Handling of intercurrent events

The intercurrent events will be handled using the treatment policy strategy, which means that the observed data will be used in the analysis regardless of whether the intercurrent events occurred.

The duration of hospitalization will be reported as a mean in each treatment condition. The treatment conditions will be compared using a difference in means and 95% CI using individual participant level data. A cross-sectional sample in each sequence period will be modelled with a GLMM with an identity link function and a Gaussian distribution to fit a linear regression model, with duration of hospitalization as the outcome and cluster (hospital) and infants who are part of the same multiple birth modelled as random effects. Treatment condition and period (before crossover vs. after crossover) will be considered fixed effects. An interaction between period and treatment will be used to evaluate whether differences between periods were affected by treatment.

The duration of hospitalization will also be summarized as a median in each of the treatment conditions. A cross-sectional sample in each sequence period will be modelled with a linear quantile mixed model, with age of final support as the outcome and cluster (hospital) and multiple birth modelled as random effects. Treatment and period (before crossover vs. after crossover) will be considered fixed effects. An interaction between period and treatment will be used to evaluate whether differences between periods were affected by treatment. This will be provided as a summary of the median (with 95% CI) in each of the treatment conditions only.

### Inter-hospital transfer due to escalating care requirements

Hospital transfer due to escalating care requirements is defined as being transferred to a hospital with a higher care capability level. Transfer from one Level 6 hospital to another Level 6 hospital is also regarded as transfer due to escalating care requirements.

This will be analyzed in the same manner as described for the primary outcome in the “Main Analysis” section, without any sensitivity or subgroup analyses.

### Death before hospital discharge

Death before discharge is anticipated to be rare given the low-risk population of infants enrolled in the trial. For this reason, the number and percentage of deaths per treatment condition, in the delivery room and prior to discharge, will be reported without any statistical inference.

### Sensitivity analyses

There are no planned sensitivity analyses for the secondary outcomes.

### Subgroup analyses

There are no planned subgroup analyses for the secondary outcomes.

## Safety outcomes

No adverse events or serious adverse events were required to be reported in this trial. This study has been deemed to be low risk.

## Data Availability

Anonymized data collected for analysis will be available 6 months after publication of the primary outcome. An application to obtain the data may be made by contacting MCTC@mcri.edu.au. No data will be released to a third-party representative without written approval by the trial sponsor.

## Abbreviations

CI: Confidence interval
CPAP: Continuous positive airway pressure
DSMC: Data and Safety Monitoring Committee
ETT: Endotracheal tube
FiO_2_: Fraction of inspired oxygen
GLMM: Generalized linear mixed model
ICC: Intracluster correlation coefficient
ILCOR: International Liaison Committee on Resuscitation
IQR: Interquartile range
IPPV: Intermittent positive pressure ventilation
QR: Quick response
SAP: Statistical analysis plan
SD: Standard deviation
SGA: Supraglottic airway
TSC: Trial Steering Committee
